# Convergence of Body-Orders in Linear Atomic Cluster Expansions

**DOI:** 10.1021/acs.jpca.5c01335

**Published:** 2025-07-28

**Authors:** Apolinario Miguel Tan, Franco Pellegrini, Stefano de Gironcoli

**Affiliations:** 19040 Scuola Internazionale Superiore di Studi Avanzati (SISSA), via Bonomea 265, 34136 Trieste, TS, Italy

## Abstract

We study the convergence of a linear atomic cluster expansion (ACE) potential with respect to its basis functions, in terms of the effective two-body interactions of elemental carbon and silicon systems. We build ACE potentials with descriptor sets truncated at body-orders *K* = 2 to *K* = 5 trained on a diverse carbon data set and on silicon dimers to pentamers. The potentials trained on diverse structures with standard ACE bases are not able to recover the correct dimer curves, much less produce stable curves or solutions. While employing ACE bases removed of self-interactions still does not generalize to the DFT-expected results, properly tailored data sets and basis sets are able to show signs of convergence and stability in the curves and expansions, suggesting a method to build potentials with interpretable bases with respect to the cluster expansion.

## Graphical abstract

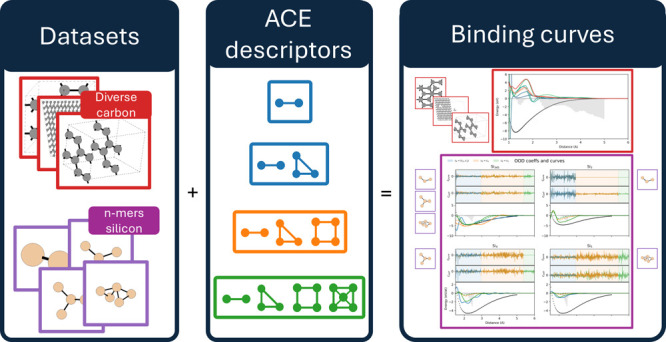

## Introduction

Decompositions of the total energy of atomic systems by *N*-body contributions, or cluster expansions,

$$\begin{array}{rl} E\left(\left\{r_{i}\right\}\right) & =V^{\left(0\right)}+\underset{i}{\sum }V^{\left(1\right)}\left(r_{i}\right)+\underset{\begin{array}{c} i,jı\neq j \end{array}}{\sum }V^{\left(2\right)}\left(r_{i},r_{j}\right)+\underset{\begin{array}{c} i,j,k\\ i\neq j\neq k\neq i \end{array}}{\sum }V^{\left(3\right)}\left(r_{i},r_{j},r_{k}\right)+\cdots \end{array}$$ 1

have been used as the framework to study material properties such as the thermodynamics of substitutional alloys^2,3^ and empirical potentials of condensed matter and molecular systems.^4–6^ While the expansion has been shown to be complete^2,3,7^ when the number of atoms matches the highest body order of the expansion, its practical effectiveness is limited to cases where the total energy converges after a few body-orders ν,^4,5,8^ as the number of terms increases combinatorially as higher orders are included.

More recently, the expansion has been used in describing local chemical environments for machine learning potentials. The underlying assumption for these descriptors is that the energy of the system is a sum of its individual atomic contributions *E*_*i*_,^9^

$$E\left(\left\{r_{i}\right\}\right)=\overset{N}{\underset{i=1}{\sum }}E_{i}\left(\left\{r_{i}\right\}\right)$$ 2

where *N* indicates the total number of atoms in the structure. The atomic energies may then be expressed as an expansion in the number of neighbors.

Improvements on the construction of *E*_*i*_ have been made in recent decades, with symmetry-respecting descriptors that use only a few terms in the expansion^9–12^ or take advantage of tensorial operations to reach an arbitrary body order.^1,13–15^ A detailed review on descriptors of local chemical environments has been done by Musil et al.^16^

A popular version of this resummation is the Atomic Cluster Expansion proposed by Drautz.^1^ In this approach, each *E*_*i*_ is expanded with a nonorthogonal basis, {*A*_ν_}, with unrestricted sums,

$$E_{i}\left(\left\{r_{i}\right\}\right)=\underset{\nu }{\sum }c_{\nu }^{\left(1\right)}A_{i,\nu }+\overset{\nu_{1}\geq \nu_{2}}{\underset{\nu_{1}\nu_{2}}{\sum }}c_{\nu_{1}\nu_{2}}^{\left(2\right)}A_{i,\nu_{1}}A_{i,\nu_{2}}+\cdots$$ 3

with single sums over neighbors

$$A_{i,\nu }=\underset{j}{\sum }f_{\nu }\left(r_{i},r_{j}\right)$$ 4

and ν indexing the terms of the expansion. The expansion has been shown to be complete,^7^ and its implementations are effective in machine learning applications.^17–21^ One of the state-of-the-art potentials, MACE,^15^ extends the formalism of ACE as a graph neural network but the interpretability of the expansion is traded for better expressivity when compared to linear potentials.

In employing interatomic potentials using the ACE framework, the selection of the basis complexity in the number of functions and maximum body-order *K* is a heuristic process and no systematic construction has been proposed yet. While ACE potentials have been shown to achieve low fitting errors, the stability of these solutions and the interpretability of the basis functions are not as studied, beyond undertakings to eliminate the self-interactions in Ho et al.^22^ and perform uncertainty quantifications in linear models in Chong et al.^23^

In the advent of foundational machine learning models^24^ as baselines training neural network potentials that do not need to be trained from scratch, it may also be worth asking if a method to systematically build a body-ordered basis with reasonable lower-order energetics can be established. In this work, we investigate the convergence of linear ACE at different body-orders *K* in terms of their projection on the lowest terms of the original cluster expansion eq 1. In particular, we consider the convergence of the potential in energy and forces on different data sets as a function of the number and type of basis functions utilized, and compare it to the stability of a real body-ordered expansion. Our study focuses on the first nontrivial term of the expansion: the two-body term. This can be easily extracted from a given potential by studying the energy profile of two isolated atoms, a dimer, as a function of distance:

$$E_{2b,i}\left(r_{i},r_{j}\right)=V^{\left(2\right)}\left(r_{i}-r_{j}\right)$$ 5

It is thus natural to ask whether this function might converge to the real dimer dissociation curve, or to another arbitrary function for a data set that does not contain dimers.

While nonlinearities can improve the performance of machine learning potentials by representing higher-order terms when the expansion is truncated, care should be taken in how they are applied as certain applications may invalidate the body-ordering of the descriptor.^14^ In the present work, we do not aim to build the most accurate potentials, but to have a more grounded understanding of their behavior in the ACE framework. As such, we limit our investigations to training simpler linear models as well as not employing very high of a body-order where returns are diminished. Linear ACE models perform comparably well relative to neural network potentials, and have been successfully employed to describe molecular^17^ and condensed phases.^18^

## Methods

### Data Sets

The data sets we use in the investigations consists of elemental structures of Carbon and Silicon. For Carbon, three data sets were used: the first set consists of 247 dimer structures at distances between 0.5 and 8 Å, with half selected for the validation set. The other two are sets of 1000 structures coming from a collection of Carbon allotropes at low pressures (0–0.1 GPa).^25^ One subset contains a homogeneous collection of diamond-like structures clustered using a Euclidean distance measure.^26^ The diamond-like data set is characterized through a histogram of distances to a reference diamond structure in Figure 1. The last subset are structures obtained using farthest point sampling^27^ to maximize its diversity. For the diamond-like and diverse data sets, a validation set was constructed from 1000 diverse structures that have not been seen in training. The Carbon data were calculated using the Quantum Espresso package^28^ using the rVV10 functional^29^ to account for nonlocal interactions.

**Figure 1:**
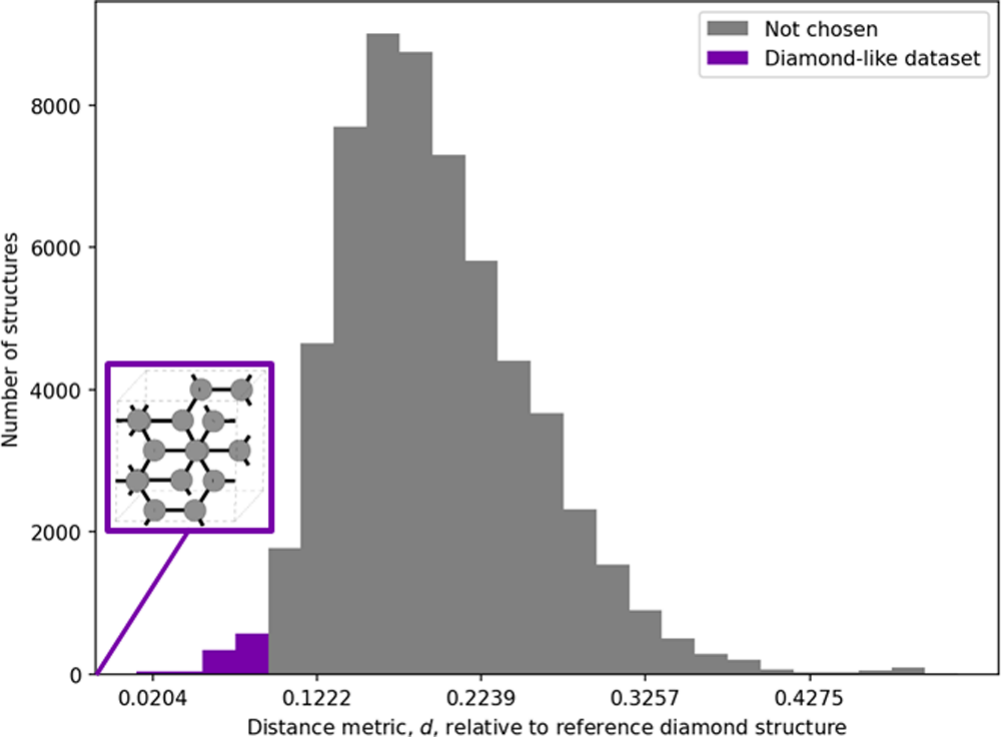
Histogram of fingerprint distances of the configurations in the Carbon data set in ref 25 relative to a reference diamond-like structure (inset). 1000 configurations were chosen within a narrow region in structure space.

Another data set of molecular Silicon structures taken from^23^ was considered as simpler cases where the maximum body order *K* in the bases is comparable to the number of neighbors in the structures. For each subset, 90*%* was used for training and the rest were used for validation.

### Descriptor

We use a linear ACE descriptor as implemented by both the pacemaker package^19^ and the ACEpotentials.jl.^21^ We start from *K* = 2 body functions and progressively add more terms of the same order. We validate the models at each addition of basis functions, and monitor when the interim model begins to overfit. Before the generalization error increases, we then introduce higher order *K* = 3 terms while keeping the number of lower order terms fixed, and then repeat the procedure for higher orders. ACE descriptors employ spherical harmonics to describe angular functions, with *n*, *l* values chosen to respect rotational symmetry. Low *n*, *l* values were chosen to avoid overfitting the model from too many basis functions.

### Fitting Procedure

Training was done with a least-squares error method for both pacemaker and ACEpotentials.jl methods. For pacemaker the loss $L_{p}$ is,

$$L_{p}=(1-\kappa )\Delta_{E}^{2}+\kappa \Delta_{F}^{2}+\Delta_{\mathrm{coeff}}+\Delta_{\mathrm{rad}}$$ 6

where Δ_E_^2^ and Δ_F_^2^ are the corresponding energy and force root-mean-square errors (RMSE), and κ is the coefficient that balances the energy and force contributions to L . The term Δ_coeff_ regularizes the basis function coefficients[Fn fn1], while Δ_rad_ regularizes the coefficients of the radial components of the basis function. ACEpotentials.jl minimizes the loss $L_{A}$ through the kernel ridge regression methods,

$$L_{A}=||W(y-\mathrm{Ac})|{|}^{2}+\lambda ||\mathrm{\Gamma c}|{|}^{2}$$ 7

where **W** encodes the relative loss weightings for energies and forces, **y** contains the labeled data, **A** is the basis matrix and **c** are the parameters to be learned to minimize $L_{A}$ . **Γ** contains a more general treatment to regularization allowing different penalizing strengths to the coefficients *c*, and λ dictates the strength of the regularization. We used the Bayesian Linear Regression to let the model dictate appropriate values for λ and **Γ**. Details of the regression methods are found in Witt et al.,^21^ and the Supporting Information discusses more details in the numerical setup. Evaluation of dimer curves were done using the Atomic Simulation Environment (ASE) by Hjorth Larsen et al.^30^

Aside from the parameters involving basis set complexities, radial bases/geometric priors, and the loss weightings, other values were set to default for both pacemaker and ACEpotentials.jl to ensure a reasonable set of controlled parameters.

## Results and Discussion

### Self-Interacting ACE Bases

We start by fitting the dimer data set with an increasing number of basis functions, *N*_f_, up to 250 elements and *K* = 4 body-order. The loss and dimer curves are shown in Figure 2, where light green, green, and dark green indicate data from potentials trained using *K* up to 2, 3, and 4, respectively. The top plot shows the dependence of the loss function L on *N*_f_. The validation loss (solid curve) converges for *K* = 2 and increases with the addition of higher order functions, jumping 2 orders of magnitude as more *K* = 4 functions are added. This is of course to be expected, as the data set consists solely of 2 body structures. The bottom plot highlights the dimer curves of the potentials trained from the dimer set at the highest *N*_f_ of a particular order *K*. We see that all potentials are able to reproduce the dimer curve as expected from the DFT results.

**Figure 2:**
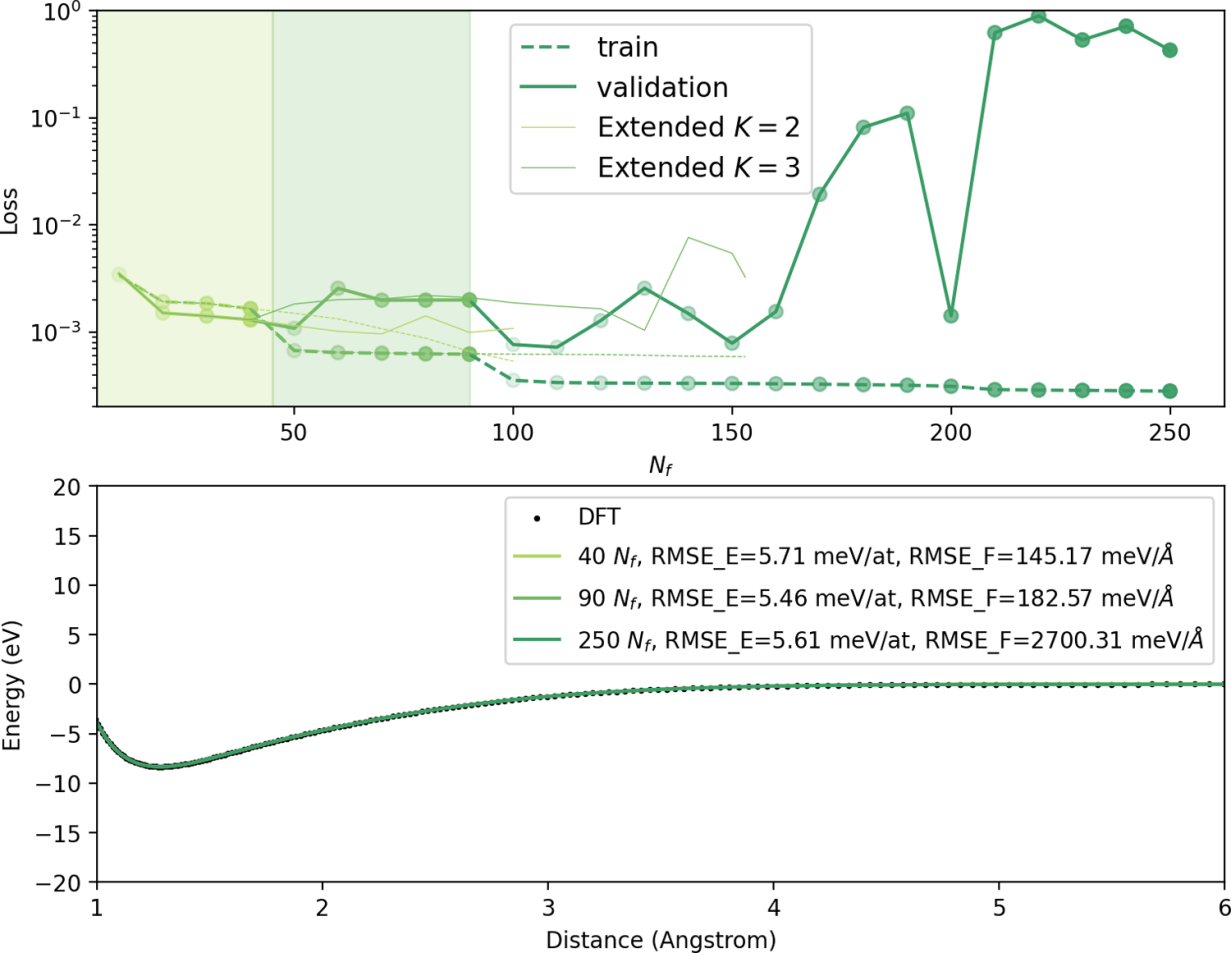
Pacemaker (Top) loss as a function of number of basis functions, *N*_f_, for the dimer data set. Training and validation errors as dashed and solid curves. Curve colors indicate potentials built from basis sets which are exclusively two-body (light green), up to three-body (green), and up to four-body functions (dark green), respectively. Light solid lines indicate the loss if more functions were introduced without introducing higher *K*. (Bottom) Dimer curves for each of the potentials trained at certain *N*_f_, with reference DFT data as points. Heavy lines represent the potential with the largest *N*_f_ before functions of higher *K* are introduced.

The energy RMSE for the representative potentials are around 5 meV/at, and force RMSE are slightly less than 200 meV/Å for *K* = 2 and *K* = 3. The force RMSEs increase to 2700 meV/Å for the overfitting potential at *N*_f_ = 250, and are due to the short-range region of the curve having energies up to almost 2 orders of magnitude higher, making the deviations from DFT results more sensitive to errors. A comparison of the forces at the short-range region may be found in the Supporting Information C.1.

The results for the homogeneous diamond-like data set are shown in Figure 3. Unlike the potentials trained from the dimer data set, the training loss from the diamond-trained potentials reduce by an order of magnitude upon the introduction of the *K* = 3 functions. The light orange line indicates that given the same *N*_*f*_, the basis set purely constructed from *K* = 2 functions does not have as pronounced of a decrease in L . We see that the validation over the diverse set has losses improving for *K* = 3, but overfitting occurs at *K* = 4. The dimer curves from these potentials show a considerable amount of variability even after the loss is converged, and they remain far from the expected dimer behavior dictated by DFT. Of course, the lack of diversity in this data set, leading to very small variations in the radial distribution function, might be part of the reason why a rather flat 2-body curve is obtained. Moreover, the overfitting for the large descriptor size could contribute to the variability of this contribution.

**Figure 3:**
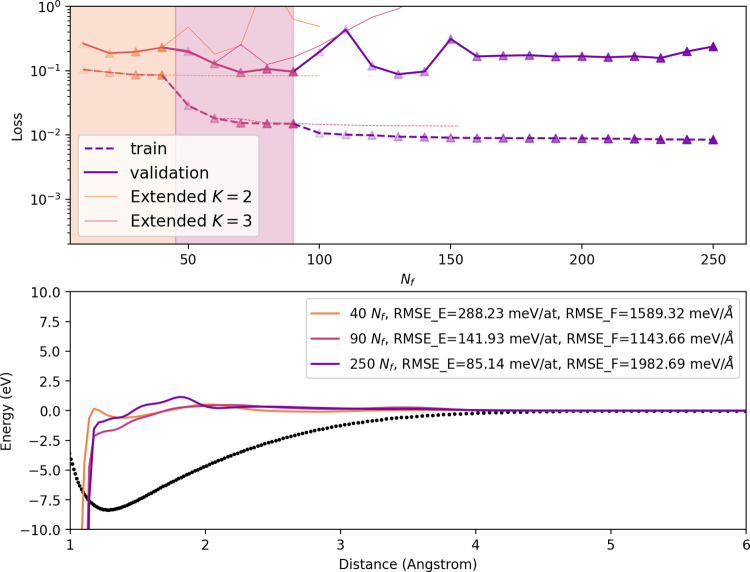
Pacemaker (top) L vs *N*_f_ and (bottom) dimer curves for the homogeneous, diamond-like data set. Orange and pink plots correspond to potentials with only *K* = 2 and up to *K* = 3 functions, respectively while purple curves include *K* = 4 functions. The *x*-values of the dimer curve start at the minimum distance found in the homogeneous data set.

We then look at the dimer curves of the potentials for different descriptor complexities, ranging from *K* = 2 to 5. The blue-green curves are potentials trained with 1000-structures while the orange-red curves are trained with a larger 50,000-structure set. Figure 4 shows these curves having higher variability compared to the ones produced by the diamond-like data set. While the curves look similar as more functions *of the same K* are introduced in the set, as seen in the *K* = 4 potentials, the curves of different *K* limits do not seem to converge even when five-body contributions are already added. The variability is much more pronounced, even among potentials with similar maximum *K* and more so when adding new terms.

**Figure 4:**
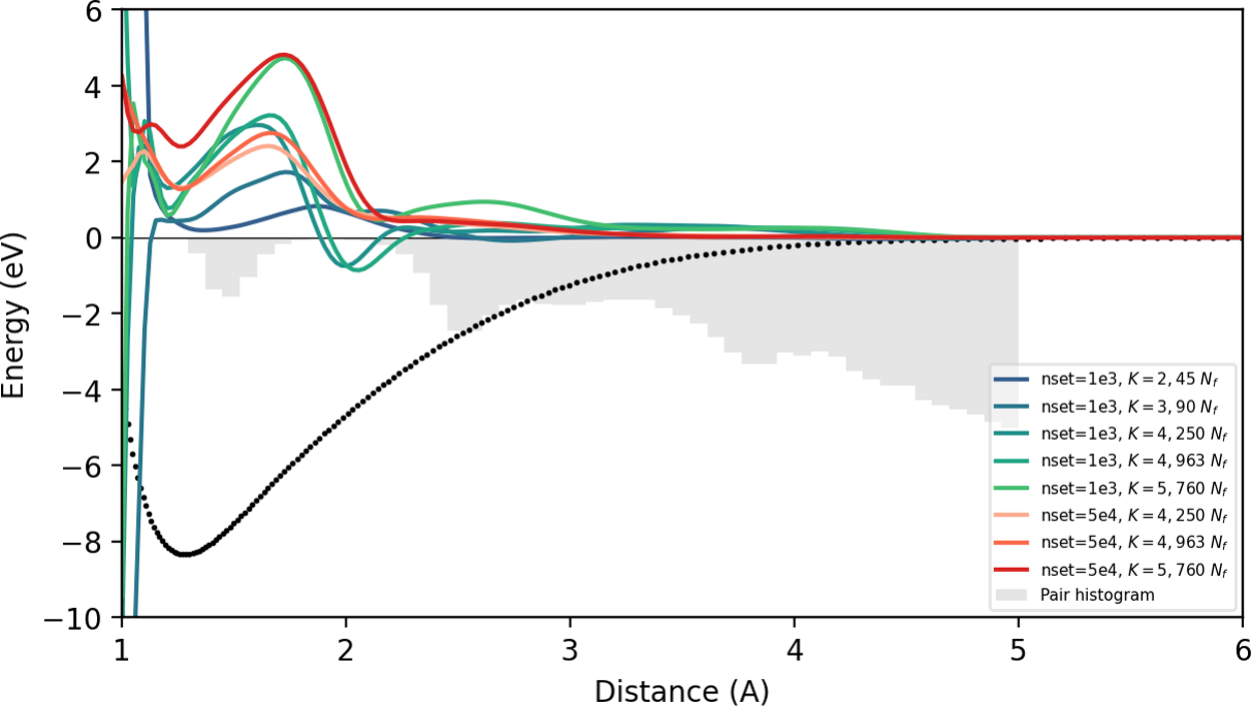
Pacemaker dimer curves for the diverse 1000-structure (blue-green) and 50,000-structure (orange-red) for various descriptor sizes. No convergence observed for both sets as higher *K* functions are introduced.

We note that the lack of pairs around the 2.0 Å region are due to the structures in the data set belonging to relatively low pressures. The comparisons between the 1000-structure and the more complete 50,000-structure data sets have virtually similar pair distribution functions due to the farthest point sampling methods done to sample the smaller subset. The pair distribution comparisons may also be seen in Supporting Information C.6.

Finally, we try the same experiment using ACEpotentials.jl using the 1000-structure set with the plots. Figure 5 shows qualitative improvements of the dimer curve in the equilibrium bond length *r*_0_ and energy minimum may be attributed to ACEpotentials.jl requiring additional user inputs (i.e., geometric priors) and more rigorous regularization schemes.^21^ The routines involving Bayesian Linear Regression also allow for the automatic selection of regularization strengths λ in eq 7 which helps reduce the number of hyperparameter sweeps done. In contrast, the absence of a more involved radial basis construction[Fn fn2] in the qualitative features of the dimer curve lead to its greater reliance on a richer data set.

**Figure 5:**
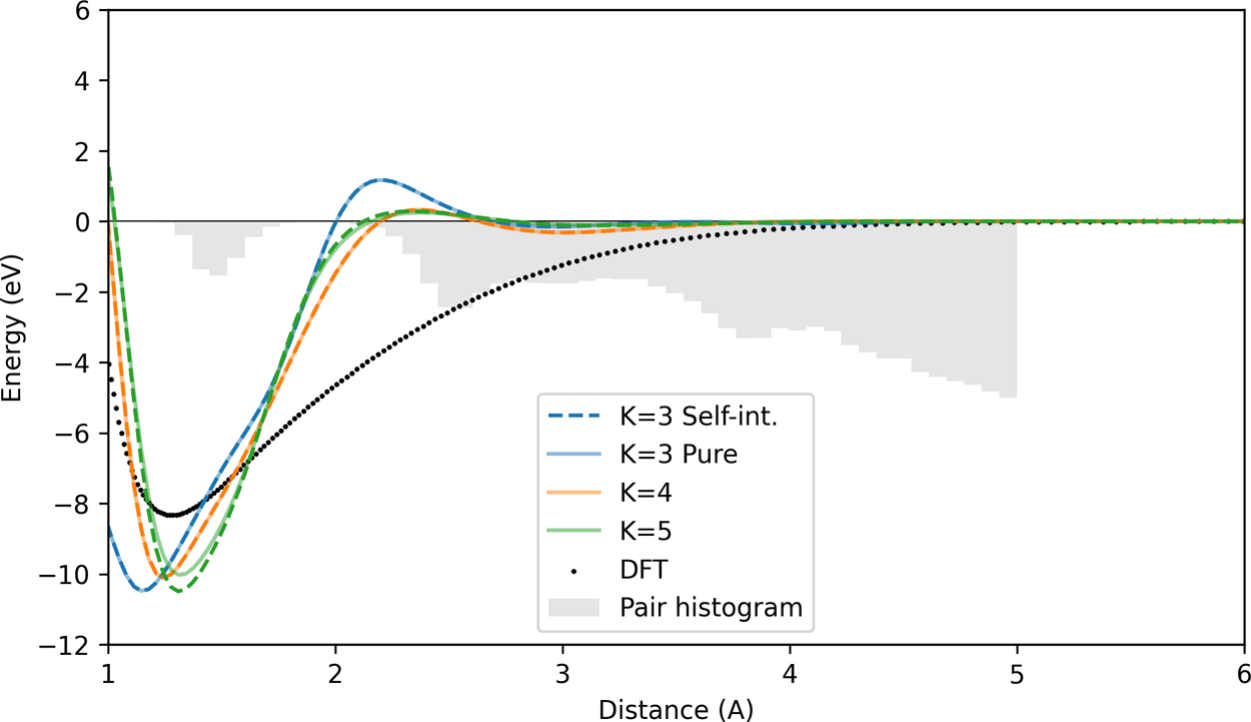
ACEpotentials.jl dimer curves for 1000-structure carbon data set trained with the Julia code.^21^ Dashed lines indicate the ACE basis (*K* = 3, 4, 5) with self-interactions similar to the one in pacemaker, while the solid lines are trained with a basis purified of self-interactions. Gray bars show the pair distance distribution in the training set. Using a purified basis for the Carbon data set yields practically identical dimer curves with those from the self-interacting basis for up to *K* = 4, with a qualitative difference in the energy well for the *K* = 5 potential.

Still, it seems that the potentials made from purified bases (solid curves) do not produce a marked improvement over the self-interacting basis (dashed lines) as they are mostly overlapping. While it seems that no improvements are seen in the purified basis, the study of the gram matrix conditioning from^22^ indicates that the potentials become less stable when the total number of relevant particles *J* (or neighbors in periodic systems) far exceed the maximum body-order, *K*, of the basis used to describe the potential[Fn fn3].

We also make sure that the performance of our linear models are reasonable by comparing their loss metrics to the performance of different neural network potentials trained on the same data set in Pellegrini et al.,^31^ as shown in Table 1. For the 1000-size subsets, we found that the linear ACE models with the most complex descriptor sets (*K* = 4, *N*_f_ = 930 and *K* = 5, *N*_f_ = 793) were able to outperform all PANNA potentials and even the *l* = 1 version of the NequIP^32^ potential. While promising, we may attribute the poor performance of the neural network potentials from the fact that 1000 structures may not be enough data and the resulting potentials may overfit without appropriate regularizations.

**Table 1:** Comparison of Linear ACE Models to Neural Network Potentials in MAE for Energies and Forces for the Carbon Dataset in Ref 25 for Datasets Containing *n*_set_ = 1000 and 50,000 Structures^a^

	*n*_set_ = 1000		*n*_set_ = 50,000	
linear potentials	*E*	*F*	*E*	*F*
pacemaker				
*K* = 4, *N*_f_ = 250	48.50	241.27	44.06	222.42
*K* = 4, *N*_f_ = 493	18.30	199.23	17.55	176.48
*K* = 4, *N*_f_ = 930	15.40	167.73	15.27	166.70
*K* = 5, *N*_f_ = 793	14.88	160.78	14.74	159.80
ACEpotentials.jl				
*K* = 3, *N*_f_ = 297	38.10	336.84		
*K* = 4, *N*_f_ = 802	18.00	210.14		
*K* = 5, *N*_f_ = 929	16.72	202.78		
neural networks				
PANNA small	22.40	248.85	17.76	162.37
PANNA mid	20.93	241.36	12.41	137.45
PANNA big	17.88	242.02	8.21	166.95
NequIP *l* = 1	20.40	213.00	7.96	110.00
NequIP *l* = 2	6.83	105.00	5.21	51.40
MACE	6.81	103.14	1.82	51.31

a Energies (*E*) are in meV/atom and force components (*F*) are in meV/Å.

However, they perform relatively worse with all other potentials when the complete 50,000-size Carbon set is used as the training set, which suggests that the nonlinearities absent in the ACE models provide a considerable improvement in training more complex data sets. Despite this, we believe that the inability of the linear models to capture the effective two-body interaction and converge to a defined shape are not due to the poor L , but rather the nature and truncation of the expansion employed in the descriptor sets, which is further discussed in the experiments with purified bases for the Silicon data set.

### Purified ACE Bases

We then repeat the same investigation on a data set of Silicon structures^23^ containing dimers, trimers, tetramers, and pentamers so the maximum number of atomic neighbors matches *K* = 5. We train potentials from subsets of 100 trimers (Si_3_), 100 tetramers (Si_4_), and 500 pentamers (Si_5_), and a mixture of the structures leaving out the dimers (Si_345_) to mimic the diverse Carbon set. Figure 6 highlights that the out-of-distribution Silicon potentials, those trained without dimers, also have curves that are not qualitatively recovered. Additionally, the Si_345_ potentials, which strikes the closest parallel to the diverse Carbon data set, exhibit the overlapping of purified and self-interacting potentials albeit at a lesser degree than the Carbon tests. When we look at the coefficients of the expansion, the nonoverlap of the lines show the instability of the solutions for the potentials at different *K*.

**Figure 6:**
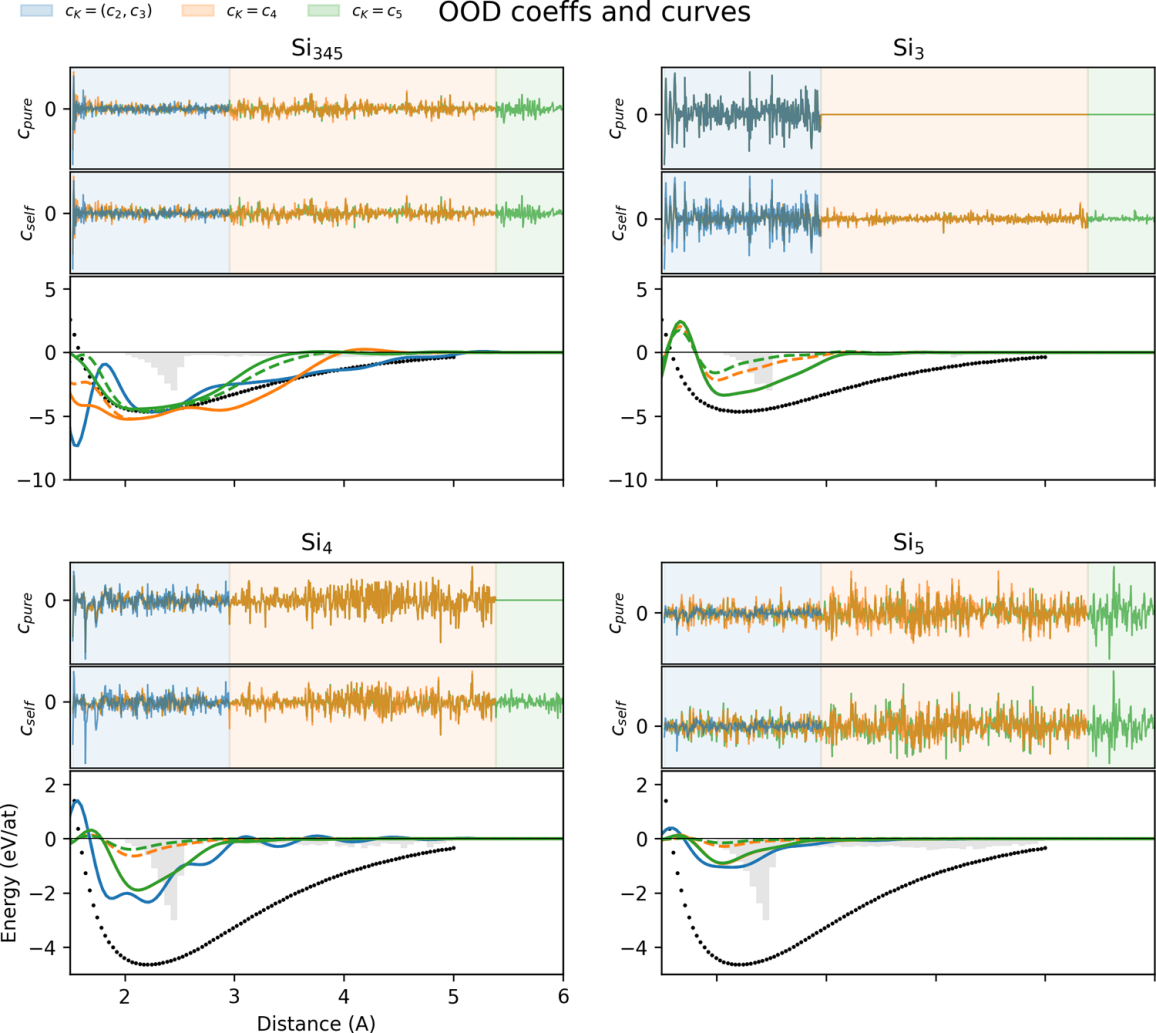
ACEpotentials.jl (bottom of each quadrant) Dimer curves of potentials from the Silicon structures in Chong et al.^23^ trained at different subsets. Si_mn_ indicate potentials were trained from data sets that include *m*-mers and *n* mers, black points show the DFT dimer energies, and gray bars indicate the distributions of pairs in the data set. Overlapping solid orange (*K* = 4) and green (*K* = 5) lines indicate that potentials with purified bases eventually converge to a defined shape when the total number of particles *N* match the maximum body order *K*. (Upper plots of each quadrant) Coefficients of the purified (*c*_pure_) and self-interacting (*c*_self_) potentials. Blue, orange, and green lines correspond to coefficients of potentials capped at *K* = 3, *K* = 4, and *K* = 5 body-ordered functions while blue, orange, and green shaded regions indicate coefficients corresponding to *K* = 2, 3, *K* = 4, and *K* = 5 body-ordered functions. Overlapping lines for a certain region mean that potentials have very similar basis coefficients, suggesting the expansion has stabilized for that particular body-order.

When focusing on the purely trimer trained Si_3_ potentials, we see signatures of convergence at the purified basis. Dimer curves of potentials at all *K* overlapping and the basis coefficients *c*_pure_ for the 2- and 3- body functions (in the blue region) are consistent with each other. The *c*_pure_ corresponding to *K* ≥ 4 functions are 0, which suggest that the self-interactions are indeed removed. In the Si_4_-trained potentials, dimer curves for the *K* = 3 basis set already deviates from the others due to the lower-order *c*_pure_ misrepresenting the higher-order tetramer interactions. We also see this happening for the Si_5_-trained potentials and even for the *K* = 4 basis sets for their misrepresentation of the 5-body interactions. Overall, the qualitative features of the dimer curves do not seem to converge as basis functions become more complex with higher *K*. This makes it difficult to apply the potentials to structures it has not seen during training.

While generalization to the correct DFT curve seems unlikely, we can still see how the coefficients of purified linear ACE models perform when doing interpolative tasks and dimers are included in the training set. It is evident in Figure 7 that the correct DFT results are recovered regardless of basis max *K* or whether the basis was purified or not. To understand whether the solution we obtain is stable, we again look at the coefficients of the potentials and see that the purification is successful in disentangling lower-order terms from the contributions of functions at higher *K*. It is also worth noting that the *c*_self_ for the Si_2_-trained data sets look identical to the purified coefficients, and is possibly due to the fact that the self-interaction terms are not needed to capture the dimer energetics.

**Figure 7:**
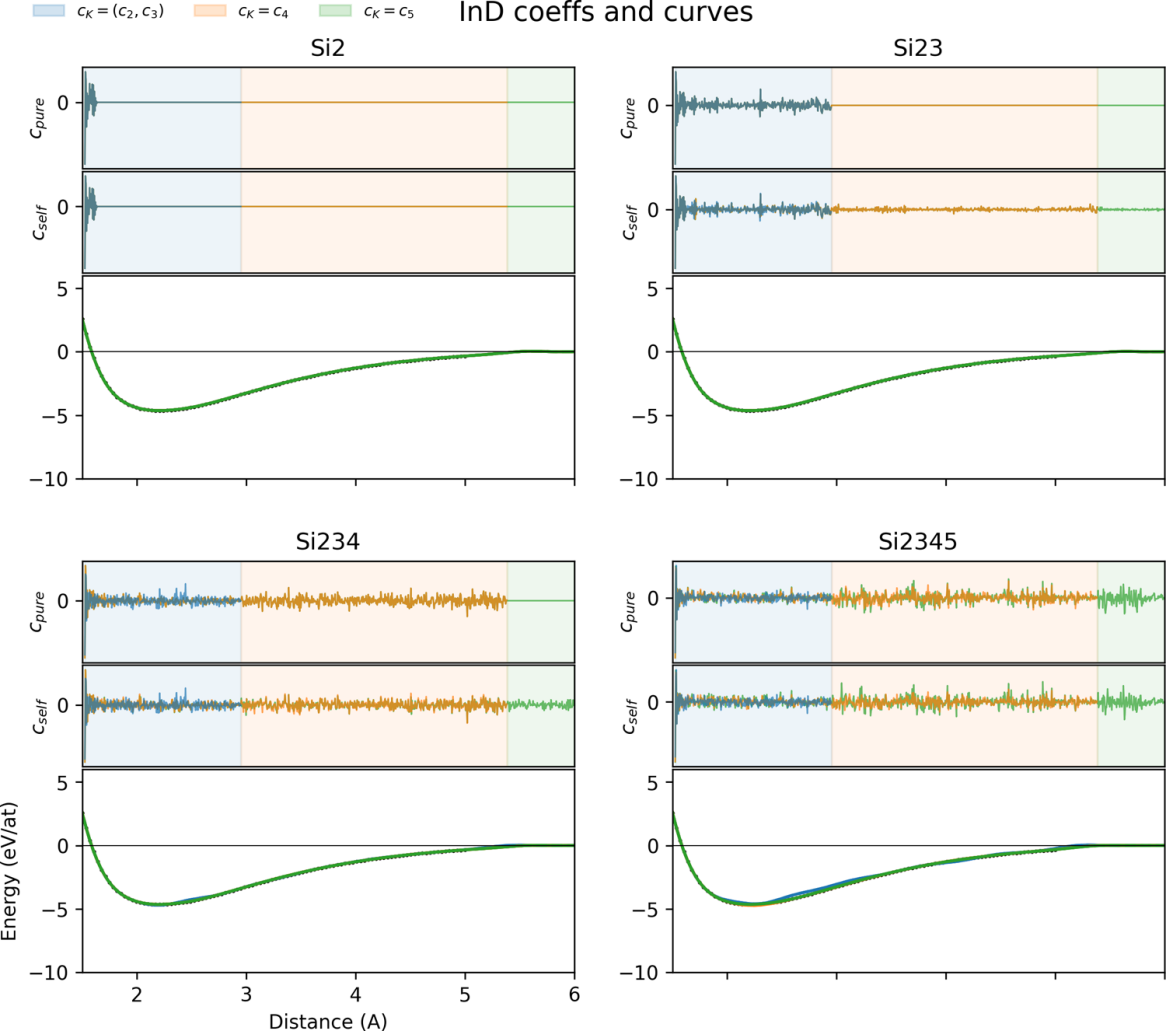
ACEpotentials.jl (top of each quadrant) ACE coefficients and (bottom of each quadrant) dimer curves of Silicon potentials trained at different subsets, including dimers in all of them. All dimer curves recover the correct DFT values, and purified potentials. All *c*_self_, except for the Si_2_ training, had higher-order renormalizations as seen in the nonzero *K* = 3, 4 for Si_23_ and *K* = 4 for Si_234_. *c*_pure_ coefficients are similar for the *K* = 2, 3 terms in Si_23_-trained potentials, and similar for *K* = 4 terms in the Si_234_ potentials.

The mismatch in the overlap of *c*_pure_ can still be seen in the *K* = 2, 3 functions of the Si_234_ sets and the *K* = 2, 3, 4 functions of the Si_2345_ sets which is the signature of aliasing that occurs when the basis is not complex enough to capture the higher-order energetics. To see if we are consistently getting not only the correct dimer curve, but a stable pair potential, we look at the *c*_pure_ of the *K* = 2 basis functions for data sets where max *K* matches the maximum number of particles *J* in Figure 8. We include the Si_2_ potential with the *K* = 3 basis as the reference coefficient set. Surprisingly, we see that no other potential was able to capture the reference coefficients completely, with greater deviations coming from the Si_234_ and Si_2345_ potentials. Looking at the discrepancy between training and validation errors between potentials, the high validation error for the Si_234_ potential suggests that more tetramers need to be sampled. For the Si_2345_ potential, both training and validation errors being high implies that the *K* = 5 basis functions as well as more pentamers are needed. Mean absolute model weights and their corresponding body-ordered features are tabulated in Supporting Information D.

**Figure 8:**
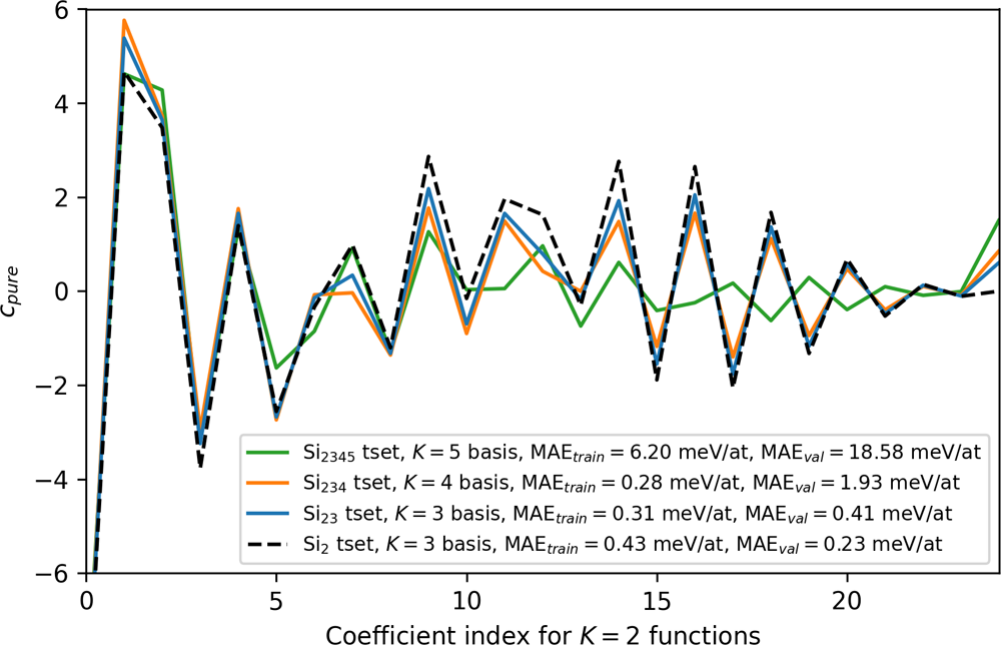
ACEpotentials.jl *c*_pure_ of the *K* = 2 basis functions for potentials with basis complexities matching the max number of particles, *K* = *J*.

## Summary and Conclusions

In this work, we studied the convergence of linear cluster expansions with respect to the body-ordered basis functions through the effective two-body interactions, *E*_2*b*_ of elemental Carbon and Silicon systems. We used the atomic cluster expansion (ACE), with descriptor sets truncated at body-orders *K* = 2 to *K* = 5 trained on a diverse Carbon data set and on Silicon dimers to pentamers. The potentials trained on diverse structures with standard ACE bases were not able to recover the correct dimer curves much less produce stable curves or solutions. While employing ACE bases removed of self-interactions still did not generalize to the DFT-expected results, properly tailored data sets and basis sets were able to show signs of convergence and stability in the curves and expansions. Stabilizing the coefficients of the expansion order-by-order by procedurally training potentials at bases up to *K* from training sets with *K*-mers, and possibly utilizing uncertainty quantification metrics as detailed in Chong et al.,^23^ shows promise as a framework to build a potential with a more interpretable basis using the cluster expansion.

## Supplementary Material



## Data Availability

Data sets and codes used for calculations may be found in https://github.com/apolmiguel/aceconverge2025

## Footnotes

1 This term includes the L1 and L2 regularizations.

2 The radial basis construction of pacemaker focuses on inner cutoffs that penalize oscillatory behavior at the short-range regions where data may be lacking, but optimizing this region of the curve is outside the study scope.

3 The notation of Ho et al. uses *N* for the body-order variable instead of *K*, and uses *J* to indicate the number of particles or neighbors considered in the system.

## Notes

Detailed information on the simulation parameters for both codes (Section A); hyperparameter investigations, particularly in balancing the loss contributions between energies and forces (Section B); additional plots (Section C), and model weights and average features of in-domain tests (Section D) (PDF)
