# Supplementary material for: Convergence of Body-Orders in Linear Atomic Cluster Expansions
Source: J Phys Chem A. 2025 Jul 28;129(31):7229–37. doi: 10.1021/acs.jpca.5c01335 (PMC12337141; doi:10.1021/acs.jpca.5c01335)
Supplement: Supplementary file 1 [file jp5c01335_si_001.pdf]

Supporting Information for: Convergence of Body-Orders in Linear Atomic Cluster Expansions

Apolinario Miguel Tan,* Franco Pellegrini, and Stefano de Gironcoli

Scuola Internazionale Superiore di Studi Avanzati (SISSA), via Bonomea 265, 34136, Trieste, TS, Italy

E-mail: atan@sissa.it

A. Simulation Parameters

For the `pacemaker` tests, the cutoff radius is set to 5 Angstrom. The potential does not use the nonlinear version of the Finnis-Sinclair potential which is set on by default in the pacemaker package. The radial basis is the Chebyshev expansion with the cosine cutoff function. The energy-force cost balancing is set that the RMSE for energies is weighted 50 times more than the forces, and further investigations is shown in Section B. The optimizer is set to BFGS with a maximum of 3000 iterations and a batch size of 100. Details about the input files may be found at the documentation of pacemaker. Table S1 distinguishes the training parameters done for the dimer curves presented in the main paper.

We used `v0.6.7` of `ACEpotentials.jl` as it was a stable version for the implementation of the purification algorithm. We used the `ACE1x.ace_basis` module to create purified bases, and is done by setting `pure = true` in the arguments. We chose to specifically control the maximum degrees per order, with the $K = 3, 4, 5$ basis sets being $[24, 20]$, $[24, 20, 16]$, and $[24, 20, 16, 12]$ respectively. These led to each basis set having 297, 802, and 929 basis func-

Table S1: Simulation Parameters for binding curve experiments in pacemaker

Training set	Descriptor label	Train steps	Validation set	nmax	lmax
Dimer 124	$K = 2, N_f = 45$	3000	val_dimer 123	100	0
	$K = 3, N_f = 90$	3000	val_dimer 123	45/8	0/2
	$K = 4, N_f = 250$	3000	val_dimer 123	45/5/4	0/2/2
Diamond 1000	$K = 2, N_f = 45$	3000	val_diverse 123	100	0
	$K = 3, N_f = 90$	3000	val_diverse 123	45/8	0/2
	$K = 4, N_f = 250$	3000	val_diverse 123	45/5/4	0/2/2
Diverse 1000	$K = 2, N_f = 45$	3000	val_diverse 1000	100	0
	$K = 3, N_f = 90$	3000	val_diverse 1000	45/8	0/2
	$K = 4, N_f = 250$	3000	val_diverse 1000	45/5/4	0/2/2
	$K = 4, N_f = 493$	3000	val_diverse 1000	45/8/4	0/3/3
	$K = 4, N_f = 960$	3000	val_diverse 1000	45/9/5	0/6/3
	$K = 4, N_f = 793$	3000	val_diverse 1000	45/8/4/3	0/3/3/2
Diverse 50000	$K = 4, N_f = 250$	3000	val_diverse 1000	45/5/4	0/2/2
	$K = 4, N_f = 493$	810	val_diverse 1000	45/8/4	0/3/3
	$K = 4, N_f = 960$	418	val_diverse 1000	45/9/5	0/6/3
	$K = 4, N_f = 793$	1151	val_diverse 1000	45/8/4/3	0/3/3/2

tions, respectively[1]. The number of basis functions were not explicitly selected as the number of functions are consequences of the maximum orders selected by the `ACE1x.ace_basis` function. We made sure to make the maximum degrees of each order even, with the lower orders having degrees considerably higher than the succeeding ones following the prescription of Ho et al.[1] to ensure that the purified bases span the same space as the original self-interacting basis. We chose `rcut=5.0` and expected equilibrium bond length `r0=1.287` for the Carbon tests as these are the expected parameters for the dataset, while `rcut=5.0, r0=2.207` were chosen for the Silicon datasets.

`ACEpotentials.jl` training was done using the Bayesian Linear Regression module by using `ACEfit.BLR()` to atuomatically determine the best values for the smoothness prior $\mathbf{\Gamma}$ and regularization strength λ. We also found out that this solver provided the most stable dimer curves with respect to changes in basis as compared to `LSQR` solvers in Witt et al.[2] as

[1]Each basis set had 24 two-body, and 273 three-body basis functions. $K = 4, 5$ had 505 four-body functions while $K = 5$ had 127 five-body functions.

seen in Fig. S1. Optimizations for LSQR-solved potentials was not done due to the vanishing dimer curves at higher K. BLR-solved potentials were used mostly "out-of-the-box" values (i.e. Cholesky factorizations used), but qualitative similarities were already present for the `ACEpotentials.jl` dimer curves. Careful hyperparameter sweeps can be done to optimize the losses, but this is not the main focus of the work.

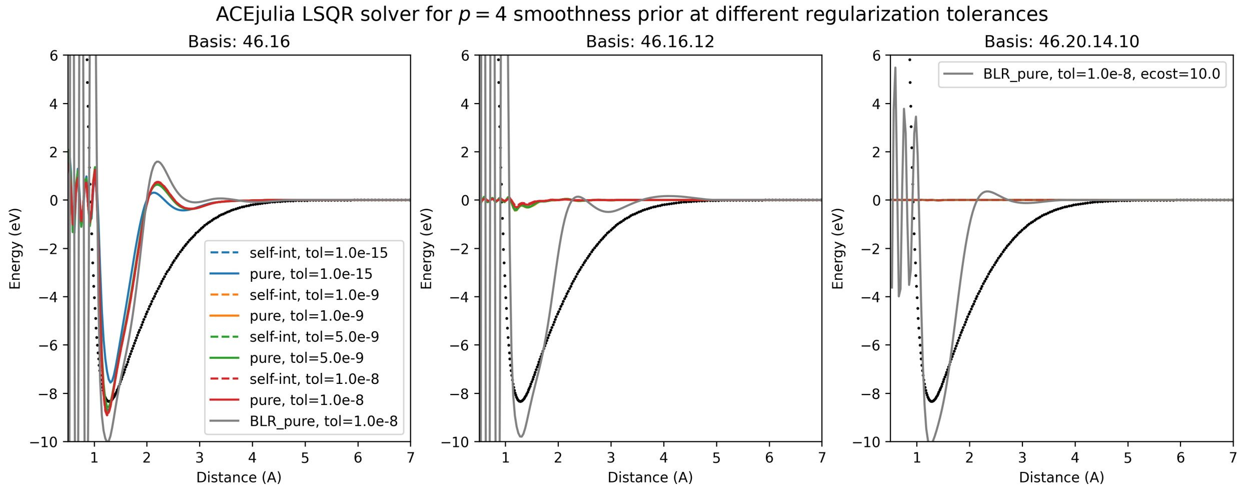

Figure S1: Comparison of ACEpotentials.jl dimer curves between LSQR (colored) and BLR (gray) solvers for basis sets $K = 3$ to $K = 5$ (left to right) and smoothness prior $p = 4$. Different colors for potentials regressed with LSQR methods indicate different regularization tolerance strengths. $K = 3$ limited LSQR-solved potentials reaches the BLR-solved potentials as the regularization tolerance is increased. $K = 4, 5$ LSQR-solved potentials had vanishing dimer curves for all tolerances. Energy-to-force loss weighting was set to 50 for all potentials except for the BLR-solved $K = 5$ which was set to 10 due to issues in getting noninvertible matrices in the solver.

B. Relative weighting for energy and force losses

In `pacemaker`, we chose default values for the regularization weights but selected the appropriate energy-to-force loss weighting ratio with the one that simultaneously minimizes the RMSE for energies and forces. Visually, it is the point closest to the origin in the right plot of Fig. S2. We see that the best results show for when the energy is weighted 50 times stronger than forces in the regression task (red), and seems to be the start of the dimer

curves being "converged" with respect to the coefficient ratio. The study focused on the energetics of the system, so weighting the energies seemed appropriate and our tests showed that increasing the coefficient from 1 to 50 had a marked improvement in the reduction of RMSE for energies, while only slightly penalizing the forces.

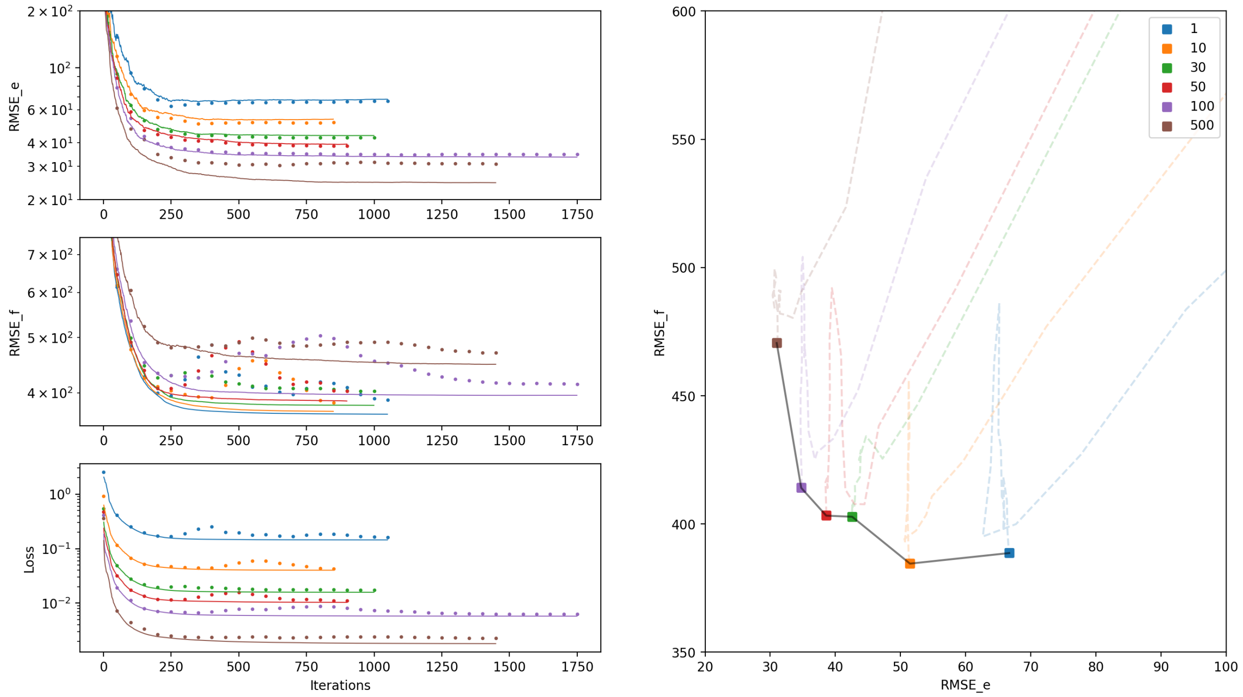

Figure S2: (Left, top to bottom) RMSE for energy, forces, and loss for different ratios for coefficients between energy and forces. 10 means energy is weighted 10 times more. (Right) RMSE forces vs. RMSE energies for the different loss coefficient ratios. Faint lines indicate the progression of each of the losses as a function of iterations. Final values are cut at the point where the validation error starts to increase considerably.

We were not able to do the same loss balancing experiments for `ACEpotentials.jl` due to problems in the matrix for the linear regression being non-invertible. We set the energy-force weighting for the Carbon dataset tests at 10, while it was set at 50 for the Silicon datasets.

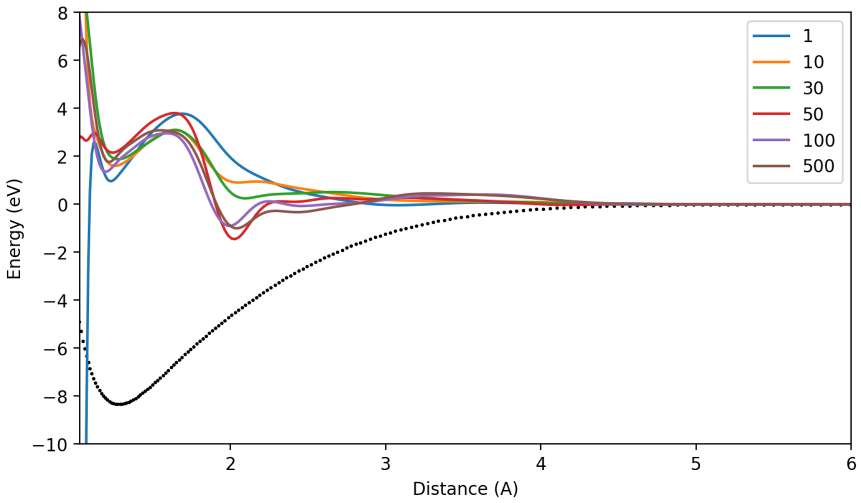

Figure S3: Dimer curves of each of the loss coefficient values at the points in the right of Fig. S2. For the remainder of the tests in the main paper, the chosen energy loss coefficient is 50 times the forces.

C. Additional plots

C.1. Force plots between DFT and dimer-trained potential

Fig. S4 shows the force values from the reference DFT data (blue scatter) as compared with the values predicted by the NNP (orange line) at the short-range region for the dimer-trained potential with $K = 4$ and $N_f = 250$. We see that despite relatively low relative errors (maximum at 13%), the absolute value of the forces admit large errors which contribute to the large validation errors at the overfitting region.

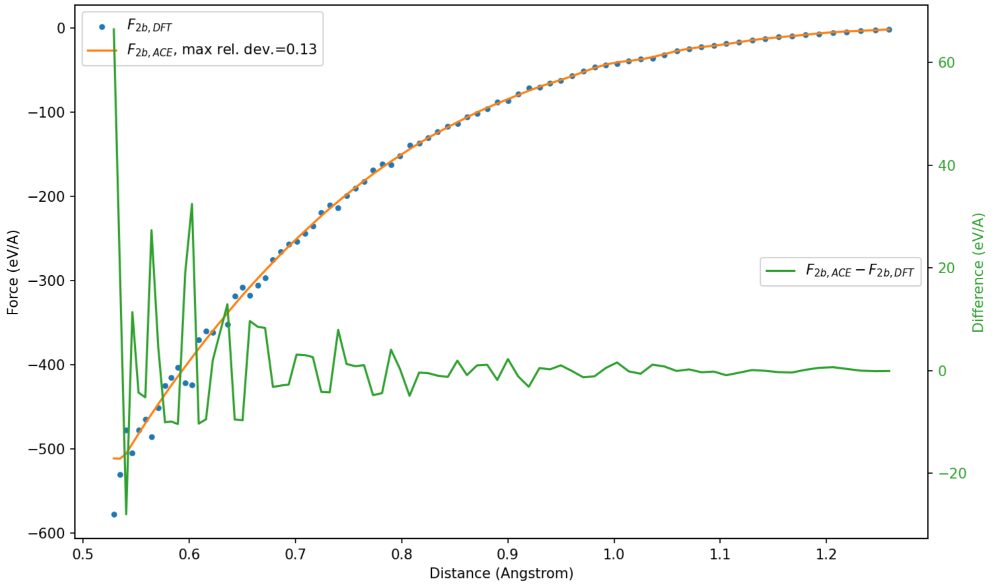

Figure S4: Forces at the short-range region for the dimer-trained potential and the difference between the forces of the DFT and the dimer-trained potential (green, right y-axis).

C.2. Loss analysis of diverse dataset dimer curves

Fig. S5 (top) shows the loss $\mathcal{L}$ vs. number of functions N_f for the potential trained on the diverse dataset and (bottom) the corresponding dimer curves at the terminal N_f for each body-order K. We see from the loss curves that opening the channel for more complex basis functions by increasing K does help improve training. Further evidence of this comes from the faint lines, which are loss data from potentials still trained with $K = 2$ (or $K = 3$) but have the same number of functions as the next higher K. Despite that, the dimer curve shows that there is a more prominent qualitative change going from $K = 2$ to $K = 3$ rather than from $K = 3$ to $K = 4$ despite having a smaller drop in losses. From here we see that the parameters at which the loss converges do not indicate that the curves have already converged.

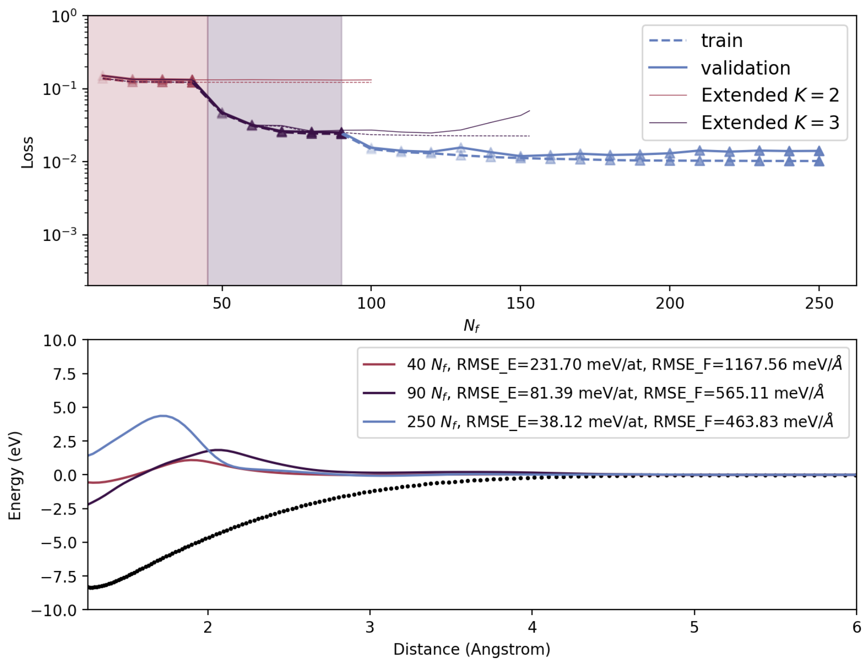

Figure S5: $\mathcal{L}$ vs. N_f (top) and dimer curves (bottom) for the diverse dataset. Orange and pink plots correspond to potentials with only $K = 2$ and up to $K = 3$ functions, respectively while purple curves include $K = 4$ functions. The x-values of the dimer curve start at the minimum distance found in the homogeneous dataset.

C.3. Biasing the diverse dataset with dimer data

Fig. S6 shows the effect of biasing the diverse dataset with an increasing number of dimer structures for the `pacemaker` tests. These tests were done to verify that the linear ACE potentials is very much capable of learning the correct interaction curve if sufficient data is provided. We show that once around 10% of the dataset is composed of dimer structures, the potential is able to approach the DFT data. We also added an additional potential trained on the dataset with 50 dimers added but initialized with a different seed to ensure that initialization effects are minimal.

Same tests were done for self-interacting and purified potentials for `ACEpotentials.jl` in Fig. S7.

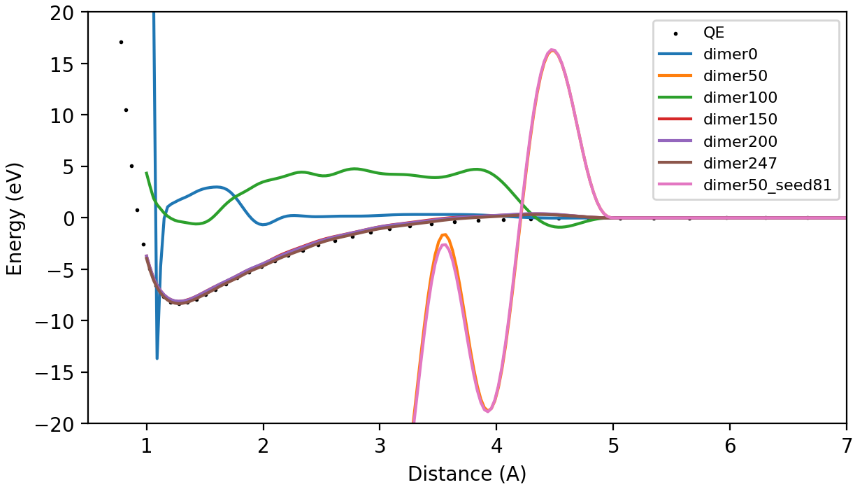

Figure S6: Dimer curves of potentials trained from the mixture of the 1000 structures from the diverse dataset with an increasing number of structures from the dimer dataset. An additional potential was also trained on the dataset with 50 dimers added but initialized with a different seed to check the initialization effects. Prominent oscillations at short-range are due to absence of bond pairs in structures in the dataset at the sub-1 Å distance region.

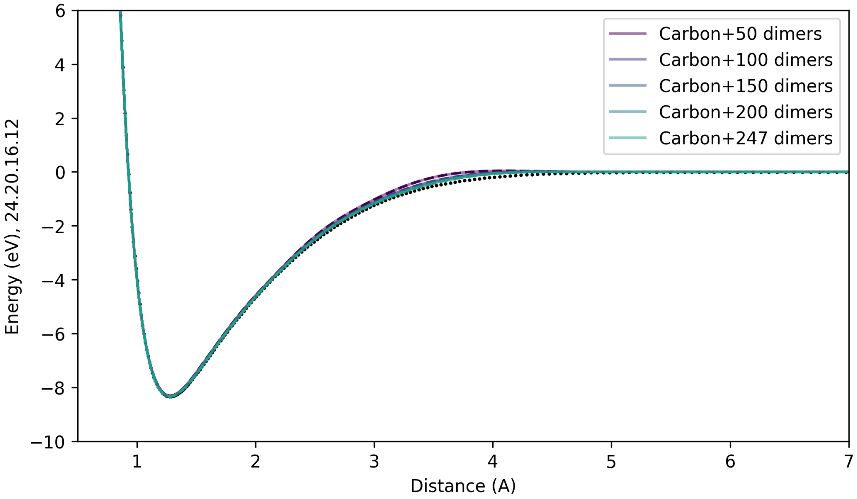

Figure S7: Dimer curves for potentials trained with dimers for carbon. For interpolative tasks we see that linear ACE potentials are able to capture the data well.

C.4. Learning curves of diverse dataset runs

Figs. S8 and S9 show the learning rates for the diverse dataset with 1000 and 50000 structures, respectively. The different colors indicate the descriptor sets of varying complexity and body-order as controlled by K and N_f. In general, we see that the potentials trained by the dataset with fewer structures approach the overfitting regions much faster and indicate that less complex descriptor sets, which have less parameters, are less susceptible to overfitting. On the other hand, the potentials trained with 50 times the data as compared previously still haven't overfit. Both learning curves have losses around the same order of magnitude, which indicates that the larger dataset benefits from the more complex descriptor sets[2].

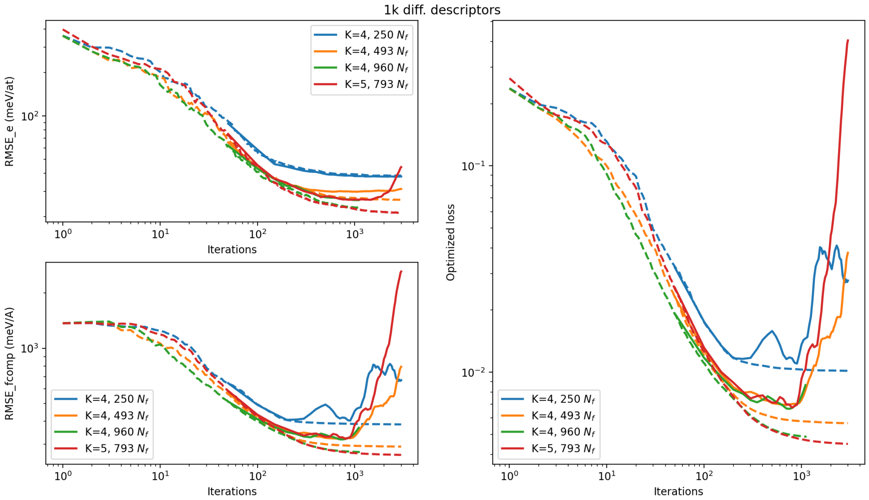

Figure S8: Learning rates for (upper left) energy, (lower left) force, and (right) total for the diverse dataset with 1000 structures. Different colors indicate different descriptor sets with varying K and N_f.

[2]We did not train for longer as it would be computationally expensive without the promise that the losses and dimer curves would have improved by then.

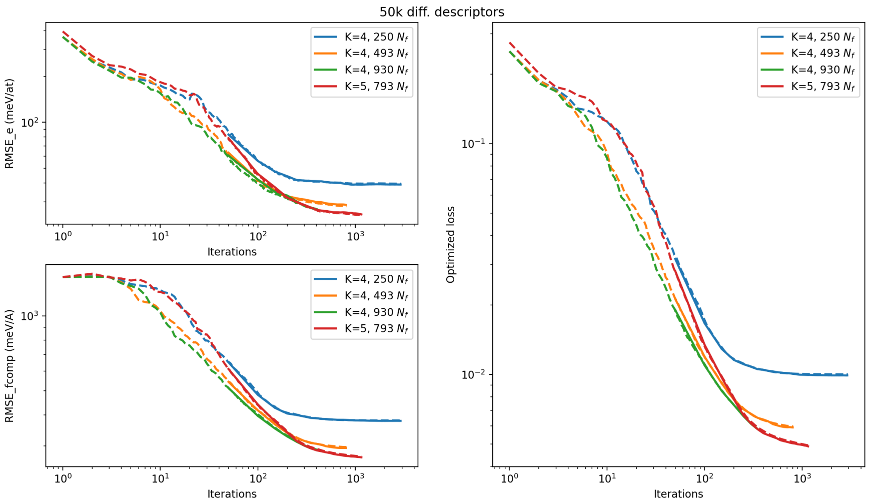

Figure S9: Learning rates for (upper left) energy, (lower left) force, and (right) total for the diverse dataset with 50000 structures.

C.5. Learning curves and dimer curves for 5k and 10k runs

Figs. S10 and S11 show the learning rates for the diverse dataset with 5000 and 10000 structures, respectively. The different colors indicate the descriptor sets of varying complexity and body-order as controlled by K and N_f. Less prominent overfitting is observed in the 5000 dataset as compared to the learning curve of the potentials trained on the 1000-structure dataset. The learning curves of the 10000 dataset do not show any signs of overfitting after the runs were completed. Their corresponding dimer curves in Fig. S12 and S13 show similar behavior from the potentials trained from the 1000 and 50000 structures in that the dimer curves do not approach a defined curve as higher K basis functions are introduced in the descriptor set.

C.6. Pair distribution comparison of 1k and 50k diverse Carbon datasets

The pair distributions for the 1k and the 50k-structure datasets in Fig. S14 are practically identical, with a lack of distances a 2.0 Å. The region may be supplemented by more varied structures (n-mers, high-pressure structures) as done in Qamar et al.[3], but a separate

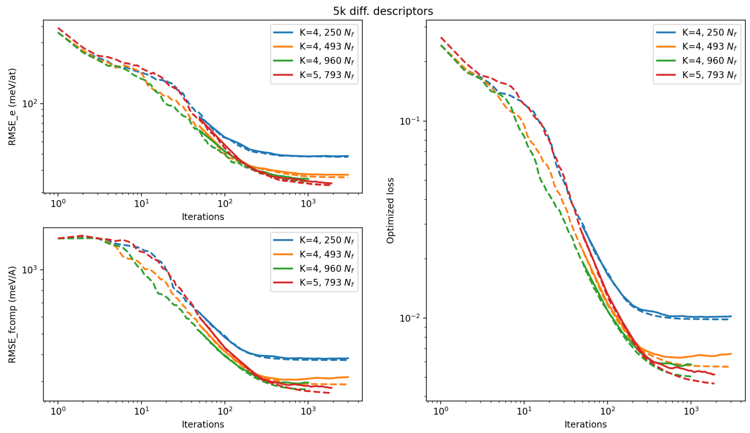

Figure S10: Learning rates for (upper left) energy, (lower left) force, and (right) total for the diverse dataset with 5000 structures. Different colors indicate different descriptor sets with varying K and N_f.

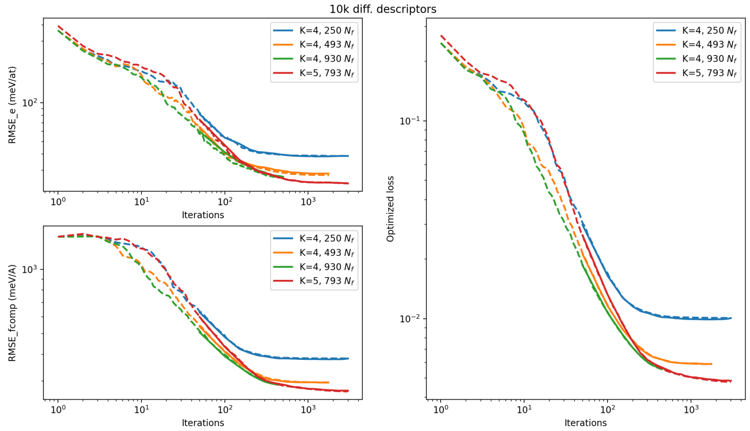

Figure S11: Learning rates for (upper left) energy, (lower left) force, and (right) total for the diverse dataset with 10000 structures.

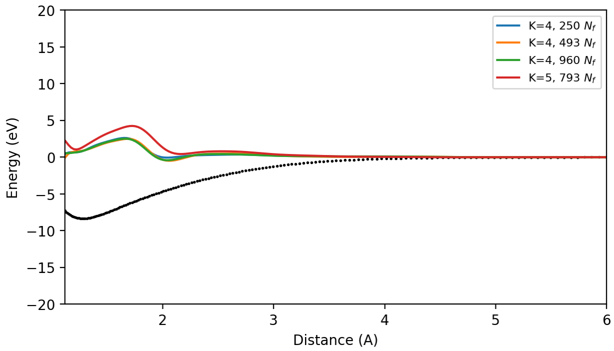

Figure S12: Dimer curves for the diverse dataset with 5000 structures. Different colors indicate different descriptor sets with varying K and N_f.

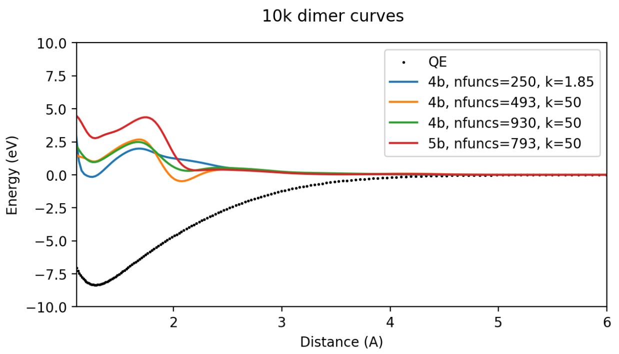

Figure S13: Dimer curves for the diverse dataset with 10000 structures. Different colors indicate different descriptor sets with varying K and N_f.

investigation must be done for this dataset.

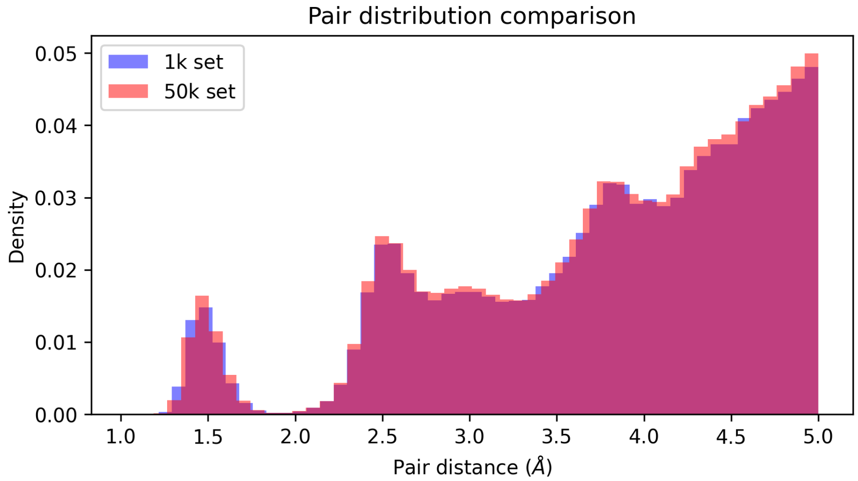

Figure S14: Pair distribution comparisons between the 1000-structure dataset and the complete 50000-structure dataset from Shaidu et al.[4].

D. Model weights and average features of in-domain tests

The mean absolute model weights, c, with respect to the features corresponding to each body-order K are tabulated in Table S2 and S3 for the purified and self-interacting bases, respectively. These results verify that potentials with purified bases have negligible coefficients for features K that have orders higher than the maximum number of atoms in the structures of the training set. Potentials with the original ACE bases will have higher or-

der K features that are relevant due to the spurious self-interactions masked as lower-order terms. The peculiar case of the Si_2-trained potentials stem from the fact that the $K = 2$ functions used (24 functions in the chosen basis) are already sufficient in capturing the pair interactions of the training set which only contains dimers. This applies to both purified and self-interacting bases, making the self-interacting basis functions practically unnecessary in capturing the correct two-body energetics.

Table S2: Mean absolute model weights for the features each K of the in-domain tests for the purified bases

Trainset	Basis	Feature			
		$K = 2$	$K = 3$	$K = 4$	$K = 5$
Si_2	24.20	2.024	$< 10^{-8}$		
	24.20.16	2.024	$< 10^{-8}$	$< 10^{-8}$	
	24.20.16.12	2.024	$< 10^{-8}$	$< 10^{-8}$	$< 10^{-8}$
Si_{23}	24.20	1.85	0.392		
	24.20.16	1.85	0.392	$< 10^{-8}$	
	24.20.16.12	1.85	0.392	$< 10^{-8}$	$< 10^{-8}$
Si_{234}	24.20	1.569	0.421		
	24.20.16	1.716	0.296	0.495	
	24.20.16.12	1.716	0.296	0.495	$< 10^{-8}$
Si_{2345}	24.20	1.429	0.315		
	24.20.16	1.420	0.291	0.434	
	24.20.16.12	1.370	0.314	0.482	0.574

References

(1) Ho, C. H.; Gutleb, T. S.; Ortner, C. Atomic Cluster Expansion without Self-Interaction. *Journal of Computational Physics* **2024**, *515*, 113271.

(2) Witt, W. C.; Van Der Oord, C.; Gelžinytė, E.; Järvinen, T.; Ross, A.; Darby, J. P.; Ho, C. H.; Baldwin, W. J.; Sachs, M.; Kermode, J.; Bernstein, N.; Csányi, G.; Ortner, C. ACEpotentials.Jl: A Julia Implementation of the Atomic Cluster Expansion. *The Journal of Chemical Physics* **2023**, *159*, 164101.

Table S3: Mean absolute model weights for the features each K of the in-domain tests for the self-interacting bases

Trainset	Basis	Feature			
		$K = 2$	$K = 3$	$K = 4$	$K = 5$
Si_2	24.20	2.024	$< 10^{-8}$		
	24.20.16	2.024	$< 10^{-8}$	$< 10^{-8}$	
	24.20.16.12	2.024	$< 10^{-8}$	$< 10^{-8}$	$< 10^{-8}$
Si_{23}	24.20	1.85	0.392		
	24.20.16	1.84	0.330	0.146	
	24.20.16.12	1.85	0.329	0.142	0.0779
Si_{234}	24.20	1.569	0.421		
	24.20.16	1.714	0.359	0.485	
	24.20.16.12	1.708	0.333	0.431	0.315
Si_{2345}	24.20	1.429	0.315		
	24.20.16	1.410	0.280	0.372	
	24.20.16.12	1.355	0.371	0.497	0.521

(3) Qamar, M.; Mrovec, M.; Lysogorskiy, Y.; Bochkarev, A.; Drautz, R. Atomic Cluster Expansion for Quantum-Accurate Large-Scale Simulations of Carbon. *Journal of Chemical Theory and Computation* **2023**, *19*, 5151–5167.

(4) Shaidu, Y.; Küçükbenli, E.; Lot, R.; Pellegrini, F.; Kaxiras, E.; De Gironcoli, S. A Systematic Approach to Generating Accurate Neural Network Potentials: The Case of Carbon. *npj Computational Materials* **2021**, *7*, 52.
